# Impact of catheter antimicrobial coating on species-specific risk of catheter colonization: a meta-analysis

**DOI:** 10.1186/2047-2994-1-40

**Published:** 2012-12-03

**Authors:** Aleksey Novikov, Manuel Y Lam, Leonard A Mermel, Anna L Casey, Tom S Elliott, Peter Nightingale

**Affiliations:** 1 Warren Alpert Medical School of Brown University, Brown, USA; 2 Department of Medicine, Warren Alpert Medical School of Brown University, Brown, USA; 3 Division of Infectious Diseases, Rhode Island Hospital, Rhode Island, 593 Eddy St., Providence, RI 02903, USA; 4 Department of Clinical Microbiology, University Hospitals Birmingham NHS Foundation Trust, Birmingham, UK; 5 Wolfson Computer Laboratory, University Hospitals Birmingham NHS Foundation Trust, Birmingham, UK

**Keywords:** Central venous catheter, Catheter colonization, Catheter-related bloodstream infection, Central line-associated bloodstream infection, Bacteremia, Antimicrobial catheter

## Abstract

**Background:**

Antimicrobial catheters have been utilized to reduce risk of catheter colonization and infection. We aimed to determine if there is a greater than expected risk of microorganism-specific colonization associated with the use of antimicrobial central venous catheters (CVCs).

**Methods:**

We performed a meta-analysis of 21 randomized, controlled trials comparing the incidence of specific bacterial and fungal species colonizing antimicrobial CVCs and standard CVCs in hospitalized patients.

**Results:**

The proportion of all colonized minocycline-rifampin CVCs found to harbor *Candida* species was greater than the proportion of all colonized standard CVCs found to have *Candida*. In comparison, the proportion of colonized chlorhexidine-silver sulfadiazine CVCs specifically colonized with *Acinetobacter* species or diphtheroids was less than the proportion of similarly colonized standard CVCs. No such differences were found with CVCs colonized with staphylococci.

**Conclusion:**

Commercially-available antimicrobial CVCs in clinical use may become colonized with distinct microbial flora probably related to their antimicrobial spectrum of activity. Some of these antimicrobial CVCs may therefore have limited additional benefit or more obvious advantages compared to standard CVCs for specific microbial pathogens. The choice of an antimicrobial CVC may be influenced by a number of clinical factors, including a previous history of colonization or infection with *Acinetobacter*, diphtheroids, or *Candida* species.

## Introduction

Central venous catheters (CVCs) have become essential in the management of critically ill patients, as well as other patient populations requiring acute or long-term medical care. Intravascular catheters can become colonized by microbial pathogens following an extraluminal or intraluminal route of endemic infection emanating from the insertion site and catheter connector/hub, respectively [1]. Meta-analyses have been published demonstrating a reduced risk of CVC colonization and CVC-related bloodstream infection with some of the currently marketed antimicrobial CVCs [2-4]. However, there are no publications that have systematically reviewed prospective, randomized clinical trials comparing antimicrobial CVCs with non-antimicrobial CVCs to determine if there are differences in the incidence of species-specific CVC colonization that might suggest a lack of efficacy for specific pathogens. We hypothesized that some currently marketed antimicrobial CVCs may lack activity against specific microbial pathogens, and as such, may select for colonization and eventual bloodstream infection caused by such pathogens. We analyzed available data to determine if there is a proven benefit at the species-specific level for use of antimicrobial CVCs and to determine if some of these CVCs may have any vulnerability in their antimicrobial spectra. We studied CVC colonization rather than CVC-related bloodstream infection since endemic CVC infections start with microbial colonization prior to the development of CVC-related bloodstream infection. Additionally, most studies have shown a 2 to 10-fold increase in episodes of CVC colonization compared to CVC-related bloodstream infection [2]. Thus, the greatest likelihood of uncovering any potential gaps in the spectrum of activity of antimicrobial CVCs would best be done by assessing CVC colonization.

## Materials and methods

We reviewed prospective, randomized clinical trials comparing antimicrobial CVCs to non-antimicrobial control CVCs. Inclusion criteria were as previously described [2]. In brief, we performed a search in Cochrane, MEDLINE, and EMBASE databases of randomized controlled trials from 1995 to current with the following strings: “central venous catheter”, “colonization”, “catheter colonization”, “bloodstream infection”, “bacteremia”, “chlorhexidine”, “benzalkonium chloride”, “rifampicin”, “minocycline”, “silver”, and “miconazole”. We chose to search from 1995 onwards as this was when the first study was published in those included in our first meta-analysis [2]. Catheter colonization was defined as at least 15 CFUs of microbial growth by semi-quantitative culture [5], or at least 1000 CFUs after quantitative vortex culture [6], or at least 100 CFUs after vortexing and sonication [6]. All statistical analyses were performed with MetAnalysis 1.0 software. Gart odds ratios (ORs) with 95% CIs were calculated for each study that met entry criteria. For each antimicrobial CVC, we analyzed the number of antimicrobial CVCs colonized with a specific microorganism or related group of microorganisms as a fraction of all colonized antimicrobial CVCs of the same type. This fraction was compared with non-antimicrobial CVCs used in the same studies analyzed in the same way.

(1) $$\begin{array}{l} \frac{Microorganism\;colonized\;control\;CVCs}{All\;colonized\;control\;CVCs}\\ \;vs.\\ \frac{Microorganism\;colonized\;test\;CVCs}{All\;colonized\;test\;CVCs} \end{array}$$

The rate of CVC colonization for each microorganism was analyzed separately and various pooled odds ratios (ORs) were calculated by both the Gart fixed-effects models (FEMs) and the DerSimonian-Laird random-effects models (REMs). The Cochran Q statistics and I^2^ test were used to assess heterogeneity. I^2^ values of 0% indicates no observed heterogeneity, whereas larger values indicated increasing heterogeneity. Results of the Gart FEM are quoted unless substantial heterogeneity was found, in which case the results of the DerSimonian-Laird REM are stated. We only included trials meeting our previously published criteria. Additionally, we only included those studies in which species-specific rates of colonization were delineated or those studies in which we were able to contact the authors directly to obtain species-specific colonization rates for antimicrobial and non-antimicrobial CVC groups. Our final analysis included an assessment for rates of CVC colonization for specific microbial species if available. If authors used the terms ‘coliforms’ or ‘diphtheroids’, we analyzed colonization rates but we did not combine these results with species-specific data. If the information regarding CVC colonization was insufficient, we attempted to contact the study corresponding author. We requested additional information from eleven authors; three authors responded before the final analysis was completed. As such, these latter studies [7-9] were included in the final analysis.

## Results

**Figure 1:**
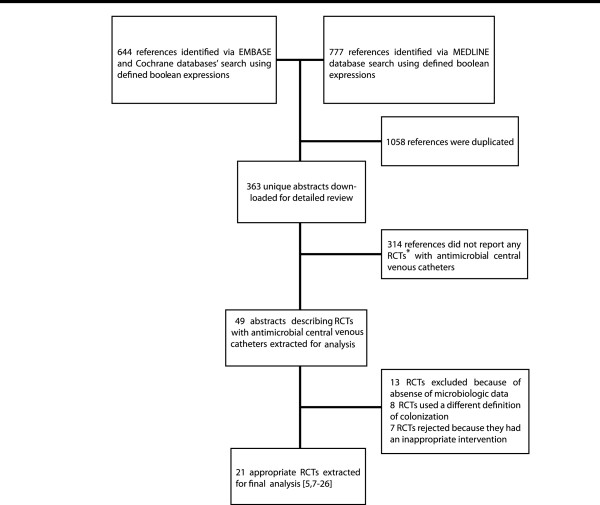
*RCT: Randomized controlled trial.

**Figure 2:**
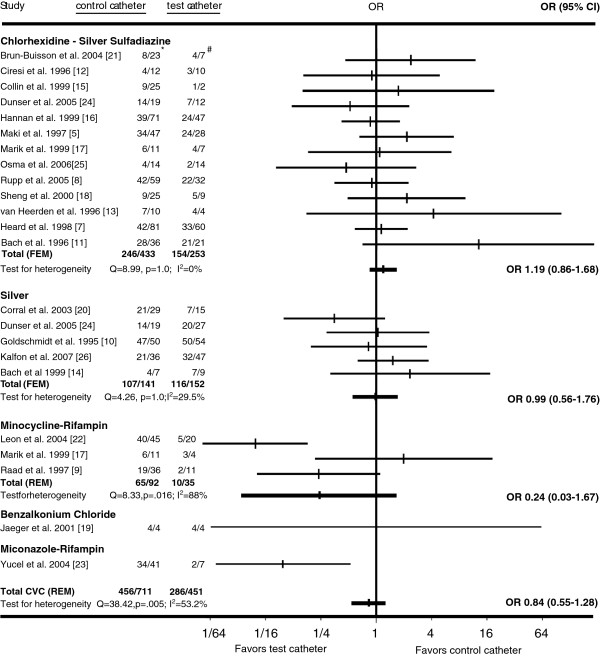
**Horizontal lines represent 95% confidence intervals (95% CI) of CVC colonization with coagulase-negative staphylococci in different trials.** They express the likelihood of test vs. control catheter colonization in relation to the vertical line that represents the null hypothesis of no difference between test and control catheters. For every type of catheter tested, the data from available trials was pooled and graphed as Gart fixed-effects model (FEM). If substantial heterogeneity was present, DerSimonian-Laird random-effects model (REM) results were used instead. *Coagulase-negative staphylococci colonized control CVCs/All colonized control CVCs. #Coagulase-negative staphylococci colonized test CVCs/All colonized test CVCs.

**Figure 3:**
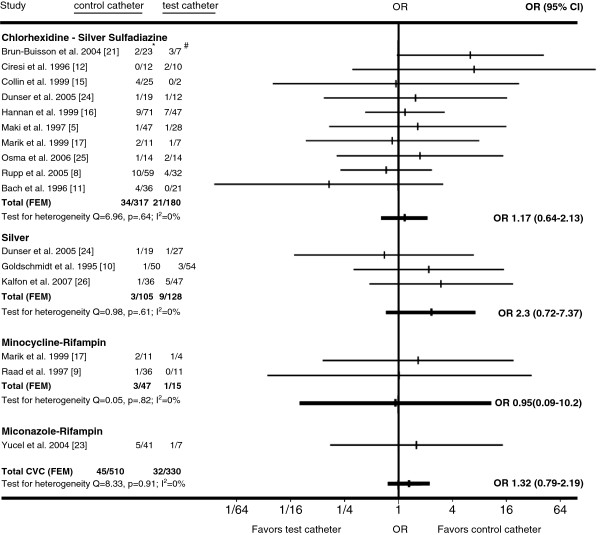
**Horizontal lines represent 95% confidence intervals (95% CI) of CVC colonization with *S. aureus *in different trials.** They express the likelihood of test vs. control catheter colonization in relation to the vertical line that represents the null hypothesis of no difference between test and control catheters. For every type of catheter tested, the data from available trials was pooled and graphed as Gart fixed-effects model (FEM). If substantial heterogeneity was present, DerSimonian-Laird random-effects model (REM) results were used instead. *S. aureus colonized control CVCs/All colonized control CVCs. #*S. aureus* colonized test CVCs/All colonized test CVCs.

Twenty-one randomized, controlled trials met our inclusion criteria [5,7-26] (Figure 1; Tables 1 and 2). Combining all antimicrobial CVCs, there was no significant difference in the proportion of colonized antimicrobial CVCs that were colonized by *Staphylococcus aureus* or coagulase-negative staphylococci compared to the corresponding fractions of colonized control CVCs (Figures 2 and 3). Similarly, the proportion of *S. aureus* or coagulase-negative staphylococci colonized chlorhexidine-silver sulfadiazine, minocycline-rifampin, or silver antimicrobial CVCs was similar to the total proportion of colonized control CVCs. The proportion of miconazole-rifampin CVCs colonized with coagulase-negative staphylococci was less than the standard CVCs. However, this reflected the results of a single study [23].

**Table 1:** Baseline characteristics of the studies used in the meta-analysis

**Study**	**CVC type**	**Study double-blinded?**	**Guidewire exchange permitted?**	**More than one study CVC permitted per patient?**	**Proportion of withdrawals or dropouts**	**Intention to treat analysis reported?**
Goldschmidt et al. 1995 [10]	Silver vs standard	--	no	yes	12.4%	no
Bach et al. 1996 [11]	CHSS vs. standard	--	no	no	--	no
Ciresi et al. 1996 [12]	CHSS vs. standard	--	yes	yes	5.4%	no
van Heerden et al. 1996 [13]	CHSS vs. standard	--	no	no	11.5%	no
Maki et al. 1997 [5]	CHSS vs. standard	yes	yes	yes	8.8%	no
Heard et al. 1998 [7]	CHSS vs. standard	--	yes	yes	15.6%	no
Bach et al 1999 [14]	Silver vs standard	--	yes	no	13%	no
Collin et al. 1999 [15]	CHSS vs. standard	--	yes	yes	2.1%	no
Hannan et al. 1999 [16]	CHSS vs. standard	no	no	yes	--	no
Marik et al. 1999 [17]	CHSS vs. standard and MR vs. standard	no	no	no	5.8%	no
Sheng et al. 2000 [18]	CHSS vs. standard	yes	no	yes	--	no
Jaeger et al. 2001 [19]	Benzalkonium chloride vs. standard	no	no	no	--	no
Corral et al. 2003 [20]	Silver vs standard	no	yes	yes	19.8%	no
Brun-Buisson et al. 2004 [21]	CHSS vs standard	yes	yes	yes	8.6%	no
Leon et al. 2004 [22]	MR vs. standard	no	no	no	21.1%	yes
Yucel et al. 2004 [23]	Miconazole-rifampicin vs. standard	no	no	no	29.4%	no
Dunser et al. 2005 [24]	Silver vs standard	no	no	no	--	no
Rupp et al. 2005 [8]	CHSS vs. standard	yes	yes	no	9.4%	yes
Osma et al. 2006 [25]	CHSS vs. standard	--	no	no	0%	yes
Kalfon et al. 2007 [26]	Silver vs. standard	no	no	yes	19.2%	no
Raad et al.1997 [9]	MR vs. standard	no	no	yes	10.7%	no

*CVC* (central venous catheter); *CHSS* (chlorhexidine - silver sulfadiazine); *MR* (minocycline-rifampin); “-” not reported.

**Table 2:** Rates of colonization of the study and control catheters

**Study**	**Mean catheter dwell time (test catheter days vs. control catheter days)**	**Number of CVC studied (test vs. control)**	**Colonization**	
			**n**	**Rate per 1000 days**
Goldschmidt et al. 1995 [10]	13.3 vs 12.7	120 vs 113	54 (45.1%) vs 50 (44.2%)	33.8 vs 34.8
Bach et al. 1996 [11]	7.8 vs 7.8	116 vs 117	21 (18.1%) vs 36 (30.8%)	23.2 vs 39.4
Ciresi et al. 1996 [12]	12.9 vs 11.5	124 vs 127	10 (10.9%) vs 12 (12.1%)	6.3 vs 8.2
van Heerden et al. 1996 [13]	6.6 vs .6.8	28 vs 26	4 (14.3%) vs 10 (38.5%)	21.6 vs 56.6
Maki et al. 1997 [5]	6 vs 6	208 vs 195	28 (13.5%) vs 47 (24.1%)	22.4 vs 40.2
Heard et al. 1998 [7]	8.5 vs 9.0	151 vs 157	60 (39.7%) vs 81 (51.6%)	46.7 vs 57.3
Bach et al 1999 [14]	4.5 vs 2.3	34 vs 33	9 (26.5%) vs 7 (21.2%)	58.8 vs 52.2
Collin et al. 1999 [15]	9.0 vs 7.3	98 vs 139	2 (2.0%) vs 25 (18%)	2.3 vs 24.6
Hannan et al. 1999 [16]	7.5 vs 7.6	174 vs 177	47 (27.2%) vs 71 (40.2%)	36.0 vs 52.8
Marik et al. 1999 [17]	6 vs 6 vs 6	36 vs 38 vs 39	7 (19.4%) vs 4 (10.5%) vs 11 (28.2%)	32.4 vs 17.5 vs 47.0
Sheng et al. 2000 [18]	9.1 vs 8.2	113 vs 122	9 (7.1%) vs 25 (20.5%)	8.8 vs 25
Jaeger et al. 2001 [19]	14.8 vs 19.3	25 vs 25	4 (16.0%) vs 4 (16.0%)	10.8 vs 8.3
Corral et al. 2003 [20]	12 vs14	103 vs 103	29 (28.2%) vs 41 (39.8%)	23.5 vs 27.7
Brun-Buisson et al. 2004 [21]	10.5 vs 12.0	188 vs 175	7 (3.7%) vs 23 (13.1%)	3.6 vs 11.0
Leon et al. 2004 [22]	10.3 vs 10.4	187 vs 180	20 (10.7%) vs 45 (25.0%)	10.4 vs 24.0
Yucel et al. 2004 [23]	7.5 vs 6.7	118 vs 105	6 (5.1%) vs 38 (36.2%)	6.8 vs 54.0
Dunser et al. 2005 [24]	9.3 vs 9.7 vs 10.7	160 vs 165 vs 160	27 (16.9%) vs 12 (7.3%) vs 19 (11.9%)	18.1 vs 7.5 vs 11.9
Rupp et al. 2005 [8]	6.9 vs. 6.7	384 vs 393	32 (9.3%) vs 59 (16.3%)	12.1 vs 22.4
Osma et al. 2006 [25]	11.7 vs 8.9	64 vs 69	14 (21.9%) vs 14 (20.3%)	18.7 vs 22.8
Kalfon et al. 2007 [26]	13.1 vs 12.9	320 vs 297	47 (14.7%) vs 36 (12.1%)	11.2 vs 9.4
Raad et al. 1997 [9]	6 vs 6	130 vs 136	11 (8·5%) vs 36 (26·5%)	14.1 vs 44.1

**Figure 4:**
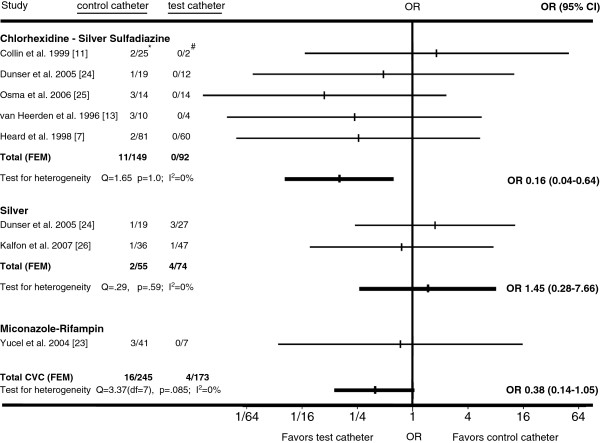
**Horizontal lines represent 95% confidence intervals (95% CI) of CVC colonization with *Acinetobacter *in different trials.** They express the likelihood of test vs. control catheter colonization in relation to the vertical line that represents the null hypothesis of no difference between test and control catheters. For every type of catheter tested, the data from available trials was pooled and graphed as Gart fixed-effects model (FEM). If substantial heterogeneity was present, DerSimonian-Laird random-effects model (REM) results were used instead. *Acinetobacter colonized control CVCs/All colonized control CVCs. #*Acinetobacter* colonized test CVCs/All colonized test CVCs.

The findings also demonstrated that there was no significant difference in the proportion of antimicrobial CVCs specifically colonized with *Acinetobacter* species compared to the proportion of *Acinetobacter* colonized control CVCs (Figure 4). Among those antimicrobial CVCs, the chlorhexidine-silver sulfadiazine CVCs colonized with *Acinetobacter* species were significantly less frequent, compared to corresponding colonized control CVCs (OR 0.16 [95%CI 0.04-0.64]). However, publication bias was detected (P=0.01).

The proportion of antimicrobial CVCs that were colonized with diphtheroids was less than that of colonized control CVCs (OR 0.45 [95%CI 0.25-0.79]). The proportion of colonized chlorhexidine-silver sulfadiazine CVCs that were colonized by diphtheroids was less than that of colonized standard control CVCs (OR 0.43 [95%CI 0.23-0.82]).

**Figure 5:**
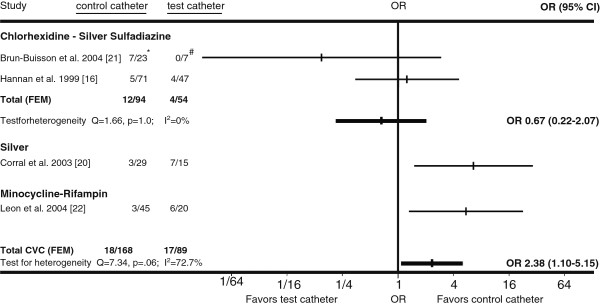
**Horizontal lines represent 95% confidence intervals (95% CI) of CVC colonization with coliform bacteria in different trials.** They express the likelihood of test vs. control catheter colonization in relation to the vertical line that represents the null hypothesis of no difference between test and control catheters. For every type of catheter tested, the data from available trials was pooled and graphed as Gart fixed-effects model (FEM). If substantial heterogeneity was present, DerSimonian-Laird random-effects model (REM) results were used instead. *Coliforms colonized control CVCs/All colonized control CVCs. #Coliforms colonized test CVCs/All colonized test CVCs.

Combining all antimicrobial CVCs, the proportion of colonized CVCs that were colonized by coliforms was greater than that of standard CVCs (OR 2.38 [95%CI 1.10-5.15]). The proportion of coliform-colonized silver CVCs and minocycline-rifampin CVCs was greater than that of standard control CVCs (Figure 5). However, these findings each represented only a single clinical trial [20,22].

**Figure 6:**
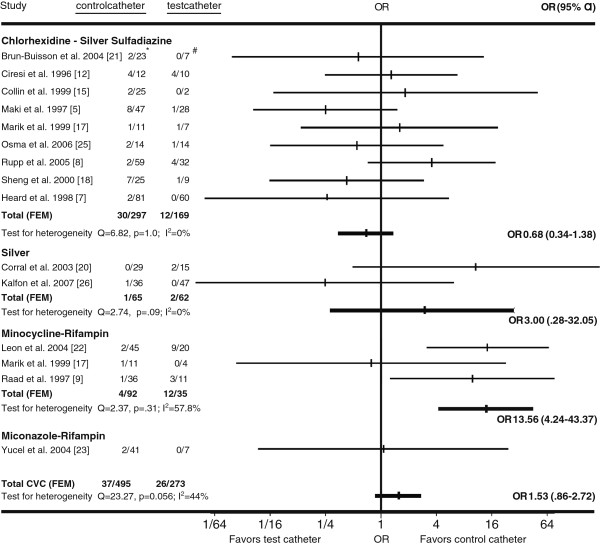
**Horizontal lines represent 95% confidence intervals (95% CI) of CVC colonization with *Candida species *in different trials.** They express the likelihood of test vs. control catheter colonization in relation to the vertical line that represents the null hypothesis of no difference between test and control catheters. For every type of catheter tested, the data from available trials was pooled and graphed as Gart fixed-effects model (FEM). If substantial heterogeneity was present, DerSimonian-Laird random-effects model (REM) results were used instead. *Candida species colonized control CVCs/All colonized control CVCs. #*Candida species* colonized test CVCs/All colonized test CVCs.

Combining all antimicrobial CVCs, there was no significant difference in the proportion of colonized antimicrobial CVCs colonized with *Candida* species compared to colonized standard CVCs (Figure 6). However, the proportion of colonized minocycline-rifampin CVCs colonized with *Candida* species was greater than that of colonized standard CVCs (OR 13.6 [95%CI 4.2-43.4]).

## Discussion

Antimicrobial-coated CVCs were developed in an effort to mitigate risk of serious CVC-related infections. Meta-analyses have shown that chlorhexidine-silver sulfadiazine CVCs, and particularly minocycline-rifampin CVCs, reduce the risk of CRBSI in prospective, randomized trials [2-4]. We compared data regarding colonization of antimicrobial and standard CVCs with known microorganisms that can cause CRBSI with an aim to investigate any potential vulnerability in the spectrum of antimicrobial activity. We found that chlorhexidine-silver sulfadiazine CVCs may have unique activity in reducing risk of colonization by *Acinetobacter* species and diphtheroids, but the former finding needs to be confirmed since we detected publication bias. Single study findings suggest that the miconazole-rifampin CVC may reduce the risk of colonization by coagulase-negative staphylococci, while the silver CVC and minocycline-rifampin CVC may be more vulnerable to coliform colonization, but these observations need to be confirmed by future studies. We identified a significant increase in the proportion of *Candida* species colonization among colonized minocycline-rifampin CVCs. Published clinical trials have not found an increased incidence of CVC-related bloodstream infections due to *Candida* species but these studies are underpowered to detect such a difference.

Our findings regarding minocycline-rifampin CVC colonization with *Candida* species, as well as coliforms, support the observations of other investigators [22,27-29]. Some investigators found significantly less microbial adherence of *Enterobacter aerogenes*, *Escherichia coli*, *Klebsiella pneumoniae*, and *C. albicans* to chlorhexidine-silver sulfadiazine CVCs compared to non-antimicrobial CVCs but no such difference when minocycline-rifampin CVCs or silver CVCs were tested [28]. Additionally, they found increased microbial adherence of *C. albicans* to minocycline-rifampin CVCs compared to control CVCs in an *in vitro* model.

Our study has important limitations. We looked at CVC colonization rather than CVC-related bloodstream infection. However, as previously stated, for endemic intravascular CVC infections, CVC colonization is a prerequisite for bloodstream infection. As such, we feel that our findings have clinical relevance. We were unable to assess differences in CVC colonization based on the anatomic site of CVC insertion as this information was unavailable in the majority of the studies included in our analysis. Another potential weakness was the variable reported use of neutralizers when cultures of antimicrobial CVCs were performed.

Prospective studies and the ensuing meta-analyses have demonstrated the attributes of antimicrobial CVCs. However, we were interested in further understanding potential unintended consequences of widespread use of such devices. The data presented herein suggest that some antimicrobial CVCs may not reduce risk of CVC infections due to *Candida* or coliforms compared to uncoated CVCs despite showing overall benefit in clinical trials. As such, clinicians should weigh potential risks and benefits when contemplating use of specific antimicrobial CVCs in patients with prior colonization or infection due to these pathogens or when the patients are located in clinical areas where these microorganisms are endemic. Novel CVCs combining components of previously studied catheters have been demonstrated to have broader antimicrobial coverage and may well be less prone to colonization with select microorganisms as demonstrated in our investigation [30].

## Competing interests

Dr. Mermel has had research funding from Astrellas and he has served as a consultant for Angiotech, Bard, Catheter Connections, Fresenius, ICU Medical, Semprus, and Teleflex. Dr. Elliott has received research funding from Carefusion, 3M and Becton Dickinson and has served on an advisory board for 3M. Drs. Novikov, Lam, Casey and Nightingale have no competing interest.

## Authors’ contributions

This project was initiated by LAM. AN, MYL, and ALC assisted in the meta-analysis; ALC and PN carried out statistical analysis; AN and MYL drafted the initial manuscript; ALC, TSE, PN, and LAM reviewed and amended the manuscript. All authors read and approved the final manuscript.
